# Erinacine A-enriched *Hericium erinaceus* mycelia promotes longevity in *Drosophila melanogaster* and aged mice

**DOI:** 10.1371/journal.pone.0217226

**Published:** 2019-05-17

**Authors:** I-Chen Li, Li-Ya Lee, Ying-Ju Chen, Ming-Yu Chou, Ming-Fu Wang, Wan-Ping Chen, Yen-Po Chen, Chin-Chu Chen

**Affiliations:** 1 Biotech Research Institute, Grape King Bio Ltd, Zhong-Li District, Taoyuan City, Taiwan; 2 Department of Food and Nutrition, Providence University, Taichung City, Taiwan; 3 Institute of Food Science and Technology, National Taiwan University, Taipei City, Taiwan; 4 Department of Food Science, Nutrition and Nutraceutical Biotechnology, Shih Chien University, Taipei City, Taiwan; 5 Department of Bioscience Technology, Chung Yuan Christian University, Zhong-Li District Taoyuan City, Taiwan; Inha University, REPUBLIC OF KOREA

## Abstract

Erinacine A-enriched *Hericium erinaceus* mycelia is a well-established potential therapeutic agent for neurodegenerative disorders. However, the effect of erinacine A-enriched *H*. *erinaceus* mycelia on promoting longevity remains unclear. This is the first study to investigate the effect of erinacine A-enriched *H*. *erinaceus* mycelia on lifespan-prolonging activity in *Drosophila melanogaster* and senescence-accelerated P8 (SAMP8) mice. Two hundred *D*. *melanogaster* and 80 SAMP8 mice of both sexes were randomly divided into four groups and were administered with either the standard, low-dose, mid-dose, or high-dose erinacine A-enriched *H*. *erinaceus* mycelia. After treatment, the lifespan was measured in *D*. *melanogaster*, and the lifespan, food intake and oxidative damage were evaluated in SAMP8 mice. Results showed that supplementation with erinacine A-enriched *H*. *erinaceus* mycelia extended the lifespan in both *D*. *melanogaster* and SAMP8 by a maximum of 32% and 23%, respectively, compared to the untreated controls. Moreover, erinacine A-enriched *H*. *erinaceus* mycelia decreased TBARS levels and induced the anti-oxidative enzyme activities of superoxide dismutase, catalase, and glutathione peroxidase. Together, these findings suggest that erinacine A-enriched *H*. *erinaceus* mycelia supplement could promote longevity, mediated partly through the induction of endogenous antioxidants enzymes.

## Introduction

Research interest in the links between diet and ageing has been growing. Studies have shown that nutrition plays a significant role in the health among the elderly, which can affect the whole process of ageing [1, 2]. In fact, several dietary supplementations with small molecules have been found to extend lifespan and prevent age-related diseases [3, 4]. These compounds comprise a large portion of natural products. From 1959 to 2017, 185 small molecules have been discovered with anti-aging activities, with 65 compounds made into clinical drugs [5]. As majority of discovered agents with longevity properties are natural products, they hold great promise in extending life expectancy in humans.

*Hericium erinaceus*, also known as Yamabushitake, Lion’s mane or Satyr’s beard, is a well-known edible and medicinal mushroom that has been used for centuries as a delicacy in several Asian countries [6]. The fruiting body and mycelia of *H*. *erinaceus* have been reported to exhibit various pharmacological actions, such as hemagglutinating [7], immunomodulatory [8], hypolipidemic [9], antihyperglycemic [10], antimicrobial [11], antitumor [12], and antioxidant [13] properties. Moreover, over the last few decades, it was discovered to have significant nootropic capabilities in the treatment of neurodegenerative diseases [14]. Furthermore, an active cyathin diterpenoida component, erinacine A, which is found only in *H*. *erinaceus* mycelia, was shown to protect against stroke, Parkinson’s disease, Alzheimer’s disease, depression, neuropathic pain, and presbycusis [15]. With these study findings demonstrating numerous health benefits, the consumption of erinacine A enriched *H*. *erinaceus* mycelia may contribute to longevity.

To date, the effect of erinacine A-enriched *H*. *erinaceus* mycelia on lifespan has not been studied. Ageing is a slow, complex process characterized by a progressive functional decline in all of the body’s cells, tissues, and organs. Since ageing occurs simultaneously in all body systems, it is unsuitable to study ageing using *in vitro* systems. Moreover, observing dynamical systems of molecules in living cells and organisms are not feasible in humans. As a result, model organisms hold the potential to reveal the physiological context of aging in humans [16]. Currently, the most common model systems that are being used in aging-related research are the budding yeast *Saccharomyces cerevisiae*, nematode worm *Caenorhabditis elegans*, fruit flies *Drosophila melanogaster*, and laboratory mice *Mus musculus* [17]. Among these, fruit flies share about 75% of disease-related genes with humans [18] while humans and mice share about 85% of gene sequences [19], which make them desirable models. As ageing research on mice is costly and time-consuming, it is tempting to study ageing using mice with reduced lifespan. The senescence-accelerated prone 8 mouse (SAMP8) has been successfully developed through the selective inbreeding of the AKR/J strain of mice donated by the Jackson laboratory in 1968 and is now increasingly used in gerontological research [20]. Therefore, in this study, the impact of erinacine A- enriched *H*. *erinaceus* mycelia on the lifespan of *D*. *melanogaster* and SAMP8 mice was investigated.

## Materials and methods

### Preparation of erinacine-enriched *Hericium erinaceus* mycelia

The *H*. *erinaceus* strain was obtained from the Bioresources Collection and Research Center in Food Industry Research and Development Institute (BCRC 35669; Hsinchu, Taiwan). The stock culture was maintained on potato dextrose agar slants at 26°C for 15 days. The seed cultures were grown in 2-L flasks containing 1.3 L of synthetic medium (0.25% yeast extract, 4.5% glucose, 0.5% soybean powder, 0.25% peptone, and 0.05% MgSO4, adjusted to pH 4.5) on a rotary shaker incubator at 120 rev/min at 25°C for 5 d. Scale-up from a shake flask to 500-L fermenters and 20-ton fermenters lasted for 5 days and 12 days, respectively. At the end of the fermentation process, the mycelia were then harvested, lyophilized, grounded to a powder, and stored in a desiccator at room temperature. 5 mg/g of erinacine A was extracted and quantified according to previous studies [21, 22].

### *D*. *melanogaster* survival test

This experiment was conducted under the standard procedure with unmated male and female flies [23]. Wild-type *D*. *melanogaster* Canton-S strains were obtained from the Bloomington Drosophila Stock Center at Indiana University (BDSC 8151; Indiana, USA). Flies enclosed within 48 h were sorted according to sex and grouped according to somatotype approximation. Eight hundred flies of each sex were randomly divided into four groups and then reared in 10 tubes containing 20 flies each. These flies were maintained in an incubator at an ambient temperature of 25°C with a 12:12 h light regime and 60% relative humidity. Media containing 5% dextrose, 5% yeast, 2% agar, and 0.23% Tegosept (Apex Bio- research Products, San Diego, USA) was used as the control group while erinacine A-enriched *H*. *erinaceus* mycelia was tested at three different concentration levels (0.11, 0.35, and 1.05 mg/mL) in the media. All dry ingredients were completely mixed with water, boiled, and then allowed to cool before dispensing. Exposure concentrations were selected based on a previous study with a minimum risk of toxicity [21]. Media was changed every 4 days, and mortality events were recorded daily.

### SAMP8 mice survival test

Eighty 6 months-old SAMP8 mice (27±5 g) of both sexes were acclimated and quarantined for 1 week prior to the initiation of the study. The animals were housed in the Modular Animal Caging System (Alternative Design, Arkansas, USA) in a well-ventilated room (10–15 air changes/h) under an ambient temperature of 25±2°C with a 12:12 h light regime and 65±5% relative humidity. The mice were randomly divided into four groups. Vehicle and three doses of erinacine A-enriched *H*. *erinaceus* mycelia (108, 215, and 431 mg/kg BW/day) were administered to the mice daily for 13 weeks by oral gavage at a dose of 10 ml/kg of body weight. Commercial chow and purified water were provided *ad libitum*. Food intake, water consumption, and change in body weight were monitored at least 2–3 times per week.

To gather maximum life span data, the animals were allowed to age and die naturally and immediately euthanized if they were found moribund. Mice were considered moribund if they fail to eat or drink, unresponsiveness to touch, or developed an ulcerated or bleeding tumor. Less than 10% of the mice were euthanized in this study, and these mice were spread among all four diet groups. Animals that were euthanized were placed in 10% formalin solution until a necropsy is performed. Date of death was recorded and used to calculate life expectancy. All animal handlings complied with guidelines set forth by the National Institutes of Health for the care and use of laboratory animals, and the protocol of this study followed the local animal ethics regulation and was approved by Providence University’s Institutional Animal Care and Use Committee (IACUC No. 20120918-A04).

### Measurement of hepatic antioxidant status

At the end of the experiment, all overnight fasted mice were anesthetized with carbon dioxide and euthanized after the liver was collected. The liver was immediately diluted by 50 mM (pH 7.0) Na_2_PO_4_ buffer, homogenized, and centrifuged to collect supernatant. The activities of thiobarbituric acid reactive substance (TBARS), superoxide dismutase (SOD), catalase (CAT), and glutathione peroxidase (GPx) were determined using commercial kits (Cayman, Michigan, USA) following the manufacturer’s instructions.

### Statistical analysis

All data are expressed as mean±SEM. Survival curves were analyzed by the Kaplan-Meier procedure with the help of Statistical Software SPSS 19.0 (SPSS, Chicago, USA). The overall differences between estimated survival curves were calculated with the log-rank test. A p-value < 0.05 was considered statistically significant.

## Results

### Effect of erinacine A-enriched *H*. *erinaceus* mycelia on *D*. *melanogaster* survival rate

**Fig 1:**
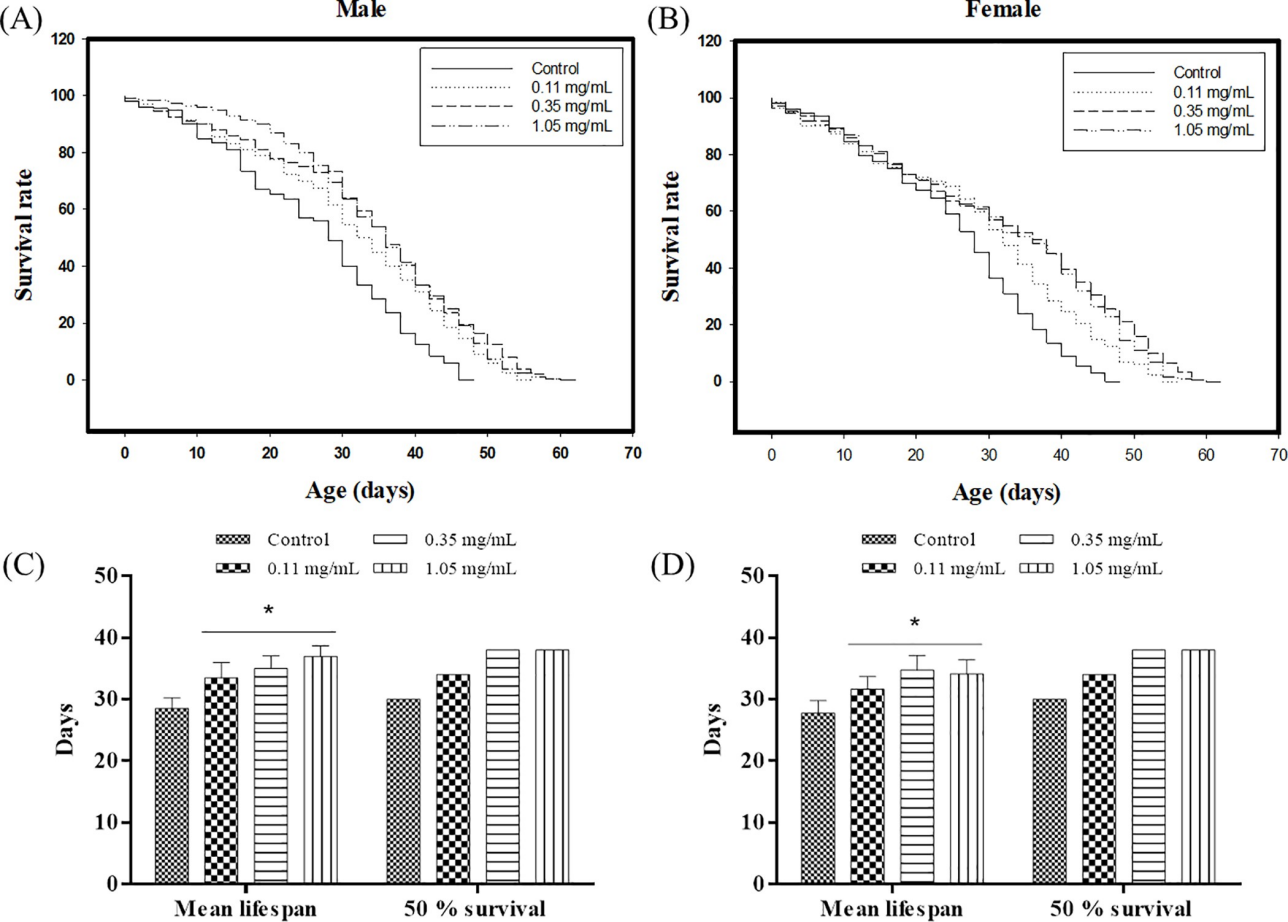
**(A, B) Maximum lifespan, (C, D) mean lifespan, and 50% survival time extension** by different concentrations of erinacine A-enriched *H*. *erinaceus* mycelia in Drosophila (A, C) male and (B, D) female flies (n = 200). *p < 0.05 compared with the control group.

The lifespan of *D*. *melanogaster* was affected by erinacine A-enriched *H*. *erinaceus* mycelia administration (Fig 1). The longest life expectancy of the male and female control groups applied with basal media were 46.9 and 45.5 days, respectively. However, when supplementing the media with erinacine A-enriched *H*. *erinaceus* mycelia, the maximum male lifespan in the 0.11 mg/mL, 0.35 mg/mL and 1.05 mg/mL application groups were found to be 53.5, 56.9, and 55.1 days, respectively (Fig 1A). Moreover, in the female population of *D*. *melanogaster* applied with erinacine A-enriched *H*. *erinaceus* mycelia, the maximum lifespan in the 0.11, 0.35, and 1.05 mg/mL dose groups were 53.1, 58.1, and 60 days, respectively (Fig 1B). The Kaplan–Meier test demonstrated that erinacine A-enriched *H*. *erinaceus* mycelia treatment could significantly extend the maximum lifespan of fruit flies in a dose-dependent manner in both sexes (*p*<0.05).

When the male flies were compared with the control groups, the mean span that was extended the most with the application of erinacine A-enriched *H*. *erinaceus* mycelia significantly increased from 28.49±0.87 days to 36.96±0.87 days with a 50% survival time (the death time of half the number of subjects) improvement from 30 days to 38 days (p < 0.05; Fig 1C and Table 1). For the females, the maximum mean lifespan with the use of erinacine A-enriched *H*. *erinaceus* mycelia significantly increased from 27.77±0.84 days to 34.05±1.15 days with a 50% survival time increase from 30 days to 38 days (p<0.05; Fig 1D and Table 1). These data demonstrated that erinacine A-enriched *H*. *erinaceus* mycelia supplementation to the media could significantly enhance the mean lifespan and 50% survival time in a dose-related manner for both male and female flies (p<0.05; Table 1).

**Table 1:** Statistical analysis of mean and median survival time in male and female Drosophila after exposure to erinacine A-enriched *H*. *erinaceus* mycelia.

**Male Drosophila**									
Groups	Number	Censored	Mean Survival Time (days)	95% CI	Median Survival Time (days)	95% CI	Log Rank p value (vs control)	Log Rank p value (vs low-dose)	Log Rank p value (vs mid-dose)
Control	200	0	28.52	[26.818–30.222]	30	[28.37–31.63]			
0.11 mg/mL	200	0	33.47	[31.518–35.422]	34	[31.48–36.52]	0.000		
0.35 mg/mL	200	0	35.75	[33.69–37.81]	38	[35.693–40.307]	0.000	0.017	
1.05 mg/mL	200	0	36.96	[35.248–38.672]	38	[35.56–40.44]	0.000	0.44	0.621
**Female Drosophila**									
Groups	Number	Censored	Mean Survival Time (days)	95% CI	Median Survival Time (days)	95% CI	Log Rank p value (vs control)	Log Rank p value (vs low-dose)	Log Rank p value (vs mid-dose)
Control	200	0	27.77	[26.122–30.222]	30	[28.16–31.84]			
0.11 mg/mL	200	0	31.63	[29.577–33.683]	34	[31.754–36.246]	0.000		
0.35 mg/mL	200	0	34.79	[32.447–37.133]	38	[33.842–42.158]	0.000	0.000	
1.05 mg/mL	200	0	34.15	[31.897–36.403]	38	[33.738–42.262]	0.000	0.005	0.235

### Effect of erinacine A-enriched *H*. *erinaceus* mycelia on SAMP8 survival rate

**Fig 2:**
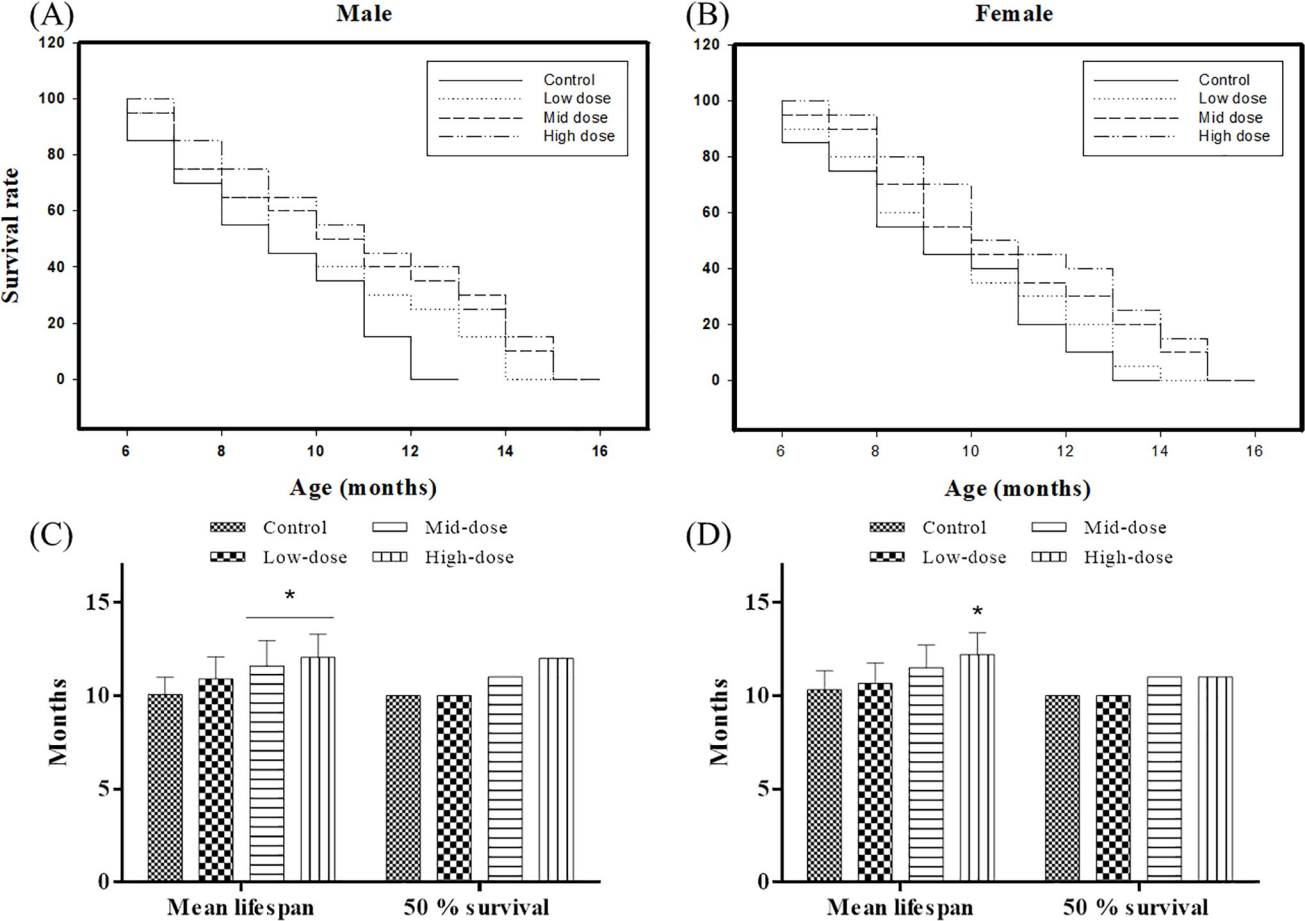
**(A, B) Maximum lifespan, (C, D) mean lifespan, and 50% survival time extension** by different concentrations of erinacine A-enriched H. erinaceus mycelia in SAMP8 (A, C) male and (B, D) female mice (n = 80). *p < 0.05 compared with the control group.

An increased life expectancy was also observed for animals applied with erinacine A-enriched *H*. *erinaceus* mycelia (Fig 2). The longest life expectancy of the male and female control mice was 13 and 14 months, respectively. However, the male low-, mid-, and high-dose erinacine A-enriched *H*. *erinaceus* mycelia SAMP8 groups showed a life expectancy of 15, 16, and 16 months, respectively (Fig 2A). Furthermore, the female SAMP8 mice fed with low-, mid-, and high-dose erinacine A-enriched *H*. *erinaceus* mycelia showed a maximum life expectancy of 15, 16, and 16 months, respectively (Fig 2B). In comparison with the control groups, both male and female SAMP8 mice fed with erinacine A-enriched *H*. *erinaceus* mycelia significantly increased their maximum life expectancy in a dose-dependent fashion (p<0.05).

Additionally, the maximum mean lifespan for male erinacine A-enriched *H*. *erinaceus* mycelia treated group was 12.05±0.62 months with a 50% survival time of 12 months, compared to 10.05±0.47 months with a 50% survival time of 10 months for SAMP8 mice with the standard control diet (Fig 2C). A similar trend was observed in the female SAMP8 mice. The maximum mean lifespan in the female mice applied with erinacine A-enriched *H*. *erinaceus* mycelia was 12.20±0.58 months with a 50% survival time of 11 months while the mean lifespan in the control group was 10.30±0.51 months with a 50% survival time of 10 months (Fig 2D). These findings suggest that erinacine A-enriched *H*. *erinaceus* mycelia can cause a statistically significant dose-dependent increase of longevity in SAMP8 mice of both sexes (p<0.05; Table 2).

**Table 2:** Statistical analysis of mean and median survival time in male and female SAMP8 mice after exposure to erinacine A-enriched *H*. *erinaceus* mycelia.

**Male Mice**									
Groups	Number	Censored	Mean Survival Time (months)	95% CI	Median Survival Time (months)	95% CI	Log Rank p value (vs control)	Log Rank p value (vs low-dose)	Log Rank p value (vs mid-dose)
Control	20	0	10.05	[9.112–10.988]	10	[7.82–12.18]			
Low-dose	20	0	10.9	[9.728–12.072]	10	[8.91–11.09]	0.135		
Mid-dose	20	0	11.6	[10.241–12.959]	11	[8.809–13.191]	0.027	0.256	
High-dose	20	0	12.05	[10.798–13.302]	12	[9.82–14.18]	0.007	0.14	0.73
**Female Mice**									
Groups	Number	Censored	Mean Survival Time (months)	95% CI	Median Survival Time (months)	95% CI	Log Rank p value (vs control)	Log Rank p value (vs low-dose)	Log Rank p value (vs mid-dose)
Control	20	0	10.3	[9.274–11.326]	10	[7.82–12.18]			
Low-dose	20	0	10.65	[9.555–11.745]	10	[8.546–11.454]	0.482		
Mid-dose	20	0	11.5	[10.289–12.711]	11	[8.82–13.18]	0.087	0.203	
High-dose	20	0	12.2	[11.04–13.36]	11	[9.247–12.753]	0.014	0.04	0.466

### Food intake and body weight

On the other hand, there were no differences in the accumulated body weight, food intake, and water consumption of SAMP8 mice fed with different regimens among the control, low-, mid-, and high-dose erinacine A-enriched *H*. *erinaceus* mycelia diet groups, verifying that erinacine A-enriched *H*. *erinaceus* mycelia did not cause any alteration in these parameters (p>0.05; Table 3).

**Table 3:** Body weights, food intakes, and water consumption in SAMP8 mice fed with different doses of erinacine A-enriched *H*. *erinaceus* mycelia for 13 weeks.

Sex	Groups	Body Weight (g)									Food Intake (g/day)			Water Consumption (mL/day)		
		Initial			Final			Gain								
Male	Control	28.40	±	0.27	31.26	±	0.30	2.85	±	0.14	5.59	±	0.05	6.32	±	0.10
	Low-dose	28.21	±	0.18	30.82	±	0.24	2.61	±	0.16	5.65	±	0.04	6.36	±	0.09
	Mid-dose	28.22	±	0.20	30.85	±	0.23	2.63	±	0.17	5.57	±	0.05	6.45	±	0.08
	High-dose	28.38	±	0.27	31.03	±	0.39	2.65	±	0.24	5.58	±	0.06	6.52	±	0.09
Female	Control	28.44	±	0.18	29.61	±	0.22	1.17	±	0.09	4.95	±	0.05	4.97	±	0.08
	Low-dose	28.69	±	0.20	29.72	±	0.23	1.03	±	0.09	5.00	±	0.04	4.97	±	0.07
	Mid-dose	28.71	±	0.25	29.80	±	0.31	1.09	±	0.15	5.02	±	0.05	4.91	±	0.06
	High-dose	28.57	±	0.14	29.53	±	0.17	0.96	±	0.11	4.96	±	0.04	4.98	±	0.08

Values were expressed as mean ± SEM and analyzed by one-way ANOVA (n = 80).

### Effect of erinacine A-enriched *H*. *erinaceus* mycelia on oxidative stress parameters

**Fig 3:**
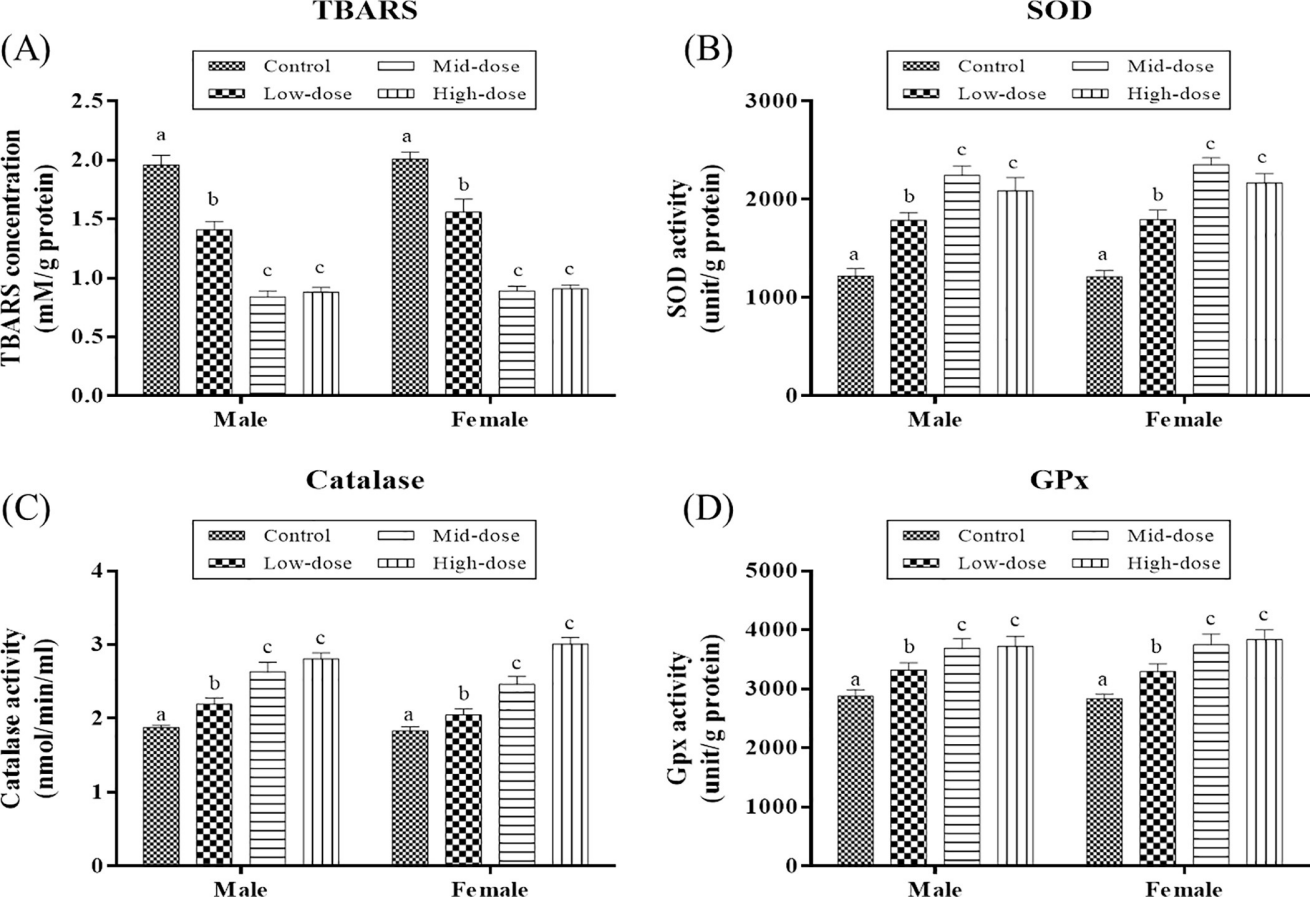
**Effect of 13 weeks of erinacine A-enriched *H*. *erinaceus* mycelia on the activities** of (A) thiobarbituric acid reactive substances (TBARS), (B) superoxide dismutase (SOD), (C) catalase, and (D) glutathione peroxidase (GPx) in the liver of SAMP8 mice (n = 80). Different letters indicate significant differences in the mean values.

Oxidative stress biomarkers such as TBARS, SOD, catalase, and GPx were assessed in livers of male and female mice exposed to erinacine A-enriched *H*. *erinaceus* mycelia. Results showed that low-, mid-, and high-dose erinacine A-enriched *H*. *erinaceus* mycelia treatment caused significant dose-dependent decreases in TBARS levels in the livers of male and female SAMP8 mice (p<0.05; Fig 3A). Moreover, significant dose-dependent elevation in other antioxidant enzyme activities (SOD, catalase, and GPx) were observed in the livers when mice of both sexes were treated with erinacine A-enriched *H*. *erinaceus* mycelia at low-, mid-, and high-doses (p<0.05; Fig 3B–3D).

## Discussion

The present study is the first report to demonstrate that erinacine A-enriched *H*. *erinaceus* mycelia possesses longevity enhancing activity in *D*. *melanogaster* and SAMP8 mice. Results clearly showed that erinacine A-enriched *H*. *erinaceus* mycelia could significantly extend the maximum lifespan, mean lifespan, and 50% survival time in a dose-dependent manner in fruit flies and SAMP8 mice of both sexes. Given that the mean life of SAMP8 mice is approximately 10 months, a 2-month increase in life expectancy is equivalent of raising the average human lifespan by 16 years [24]. Although the underlying mechanisms by which erinacine A-enriched *H*. *erinaceus* mycelia extends the lifespan of both fruit flies and SAMP8 remain poorly understood, several studies have suggested some possibilities.

One possible mechanism is potentially related to the regulation of oxidative stress-related signaling by erinacine A-enriched *H*. *erinaceus* mycelia. A number of signaling pathways such as mitogen-activated protein kinases (MAPKs) and phosphoinositide 3 kinase/Akt (PI3K/Akt) have known to be associated with cellular responses to oxidative stress [25], which play an important role in biological senescence [26]. Nevertheless, experimental evidence has shown that extension of lifespan could be obtained by increasing the antioxidant defense as well as decreasing the reactive oxygen species production [27]. In the present study, the HPLC analysis showed that erinacine A-enriched *H*. *erinaceus* mycelia contained 5 mg/g erinacine A. Interestingly, erinacine A was recently reported to exert its antioxidant activity via inducible NO synthase (iNOS)/p38 MAPK/ CCAAT/enhancer-binding protein (C/EBP) homologous protein (CHOP) pathway in an animal model of ischemic stroke [28], Jun N-terminal kinase (JNK)/p38 MAPK/nuclear factor-κB (NF-κB)/CHOP/Fas/Bax pathway in an animal model of Parkinson’s Disease [29], and PI3K/Akt/glycogen synthase kinase-3β (GSK-3β) in an animal model of depression [30]. In this regard, erinacine A-enriched *H*. *erinaceus* mycelia may act as an antioxidant upon various oxidative stress conditions and can consequently extend lifespan.

The second mechanism by which erinacine A-enriched *H*. *erinaceus* mycelia prolonged the lifespans of fruit flies and SAMP8 mice may be mediated by the induction of endogenous antioxidants enzyme activities. Results showed a significant dose-dependent elevation of SOD, catalase, and GPx activities in the livers of mice administrated with erinacine A-enriched *H*. *erinaceus* mycelia when compared to the control group. This enhanced activation of SOD enzyme activity in the liver after the administration of erinacine A-enriched *H*. *erinaceus* mycelia may be a consequence of increased endogenous enzyme synthesis or antioxidant utilization. As a result, a reduced superoxide anion radical accumulation with oxidative stress may be associated with these increased SOD activities in liver tissues and contribute to decreased liver toxicity [31]. Moreover, in human cells, SOD enzymes work in conjunction with H_2_O_2_-removing enzymes such as catalase and GPx [32]. The relative GPx and catalase protein expressions have been found to exhibit a profound decline in rats with serious oxidative injury [33]. The observed increase in catalase and GPx activities after the administration of erinacine A-enriched *H*. *erinaceus* mycelia indicated an elevated capacity to scavenge hydrogen peroxide produced in the liver. In fact, treatment with erinacine A-enriched *H*. *erinaceus* mycelia has reversed TBARS buildup in the liver of SAMP8 mice, implying that oxidative stress and reactive oxygen species (ROS) formation are reduced. In this regard, erinacine A-enriched *H*. *erinaceus* mycelia offered protection as evidenced by decreased TBARS and increased antioxidant enzyme activity, which contributed to decreased free radical generation and increased antioxidant defenses. However, further studies are warranted to examine TBARS and antioxidant activities in other organs and tissues including the brain to support this observation.

Interestingly, a number of studies have also found that reduced levels of oxidative stress in long-lived organisms not only resulted in extended lifespans but also accumulated less damage than short-lived organisms [34–36]. More than fifty years ago, the mitochondria free radical theory of aging postulated that the determinant of lifespan and many pathologies resulted from accumulating ROS produced by the mitochondria [37]. To date, several lines of evidence have corroborated this theory. One of the most direct experimental evidence was shown in transgenic mice overexpressing human catalase localized to the mitochondria, which caused a significant median and maximal lifespan extension [38]. Furthermore, catalase specifically targeted to the mitochondria was shown to have protection against some diseases such as cardiac diseases, cancer, and insulin resistance in mice [39]. This indicates that antioxidants targeting the mitochondria may not only be beneficial for life but also for health promotion. In fact, there has also been growing evidence for the benefits of erinacine A-enriched *H*. *erinaceus* mycelia to counteract age-related diseases such as cancer and neurodegenerative diseases [15, 40]. Since the impact of erinacine A-enriched *H*. *erinaceus* mycelia supplement on mitochondria function has not been well-investigated, this could provide an interesting direction for future research.

Last but not least, experiments in various models have shown that modulation of calorie intake and metabolism are important factors affecting lifespan [41–43]. Caloric restriction can result in reduced adiposity, increase gene expressions involved in fat turnover, and decrease gene expressions of inflammatory markers [44]. However, in this study, no significant differences were found in the body weight, feed intake, and water consumption in mice among the treatment groups, suggesting that erinacine A-enriched *H*. *erinaceus* mycelia enhancement of flies and mouse survival is not a consequence of reduced food intake. These results also suggested that erinacine A-enriched *H*. *erinaceus* mycelia may not cause any detrimental effects for human consumption, which is consistent with findings from previous studies [21, 22, 45]. As a result, it can be concluded that erinacine A-enriched *H*. *erinaceus* mycelia can be developed as an effective intervention to promote lifespan in mammals, including humans.

## Conclusion

This study demonstrates that erinacine A-enriched *H*. *erinaceus* mycelia can be a candidate to prolong life expectancy by reducing oxidative stress and increasing antioxidant defenses. However, further biochemical investigations of the mycelia constituents may be needed to assess the efficacy and the underlying mechanism of its action for the elongation of lifespan.

## Abbreviations

CAT: catalase
GPx: glutathione peroxidase
SAMP8: senescence-accelerated P8
SOD: superoxide dismutase
TBARS: thiobarbituric acid reactive substance

## References

[1] Ahmed T, Haboubi N. Assessment and management of nutrition in older people and its importance to health. Clinical interventions in aging. 2010;5:207–16. . 20711440 10.2147/cia.s9664 PMC2920201

[2] Leslie W, Hankey C. Aging, Nutritional Status and Health. Healthcare (Basel, Switzerland). 2015;3(3):648–58. 10.3390/healthcare3030648 . 27417787 PMC4939559

[3] Martin-Montalvo A, Mercken EM, Mitchell SJ, Palacios HH, Mote PL, Scheibye-Knudsen M, et al Metformin improves healthspan and lifespan in mice. Nature communications. 2013;4:2192 Epub 2013/08/01. 10.1038/ncomms3192 ; PubMed Central PMCID: PMCPmc3736576. 23900241 PMC3736576

[4] Harrison DE, Strong R, Sharp ZD, Nelson JF, Astle CM, Flurkey K, et al Rapamycin fed late in life extends lifespan in genetically heterogeneous mice. Nature. 2009;460(7253):392–5. Epub 07/08. 10.1038/nature08221 . 19587680 PMC2786175

[5] Ding A-J, Zheng S-Q, Huang X-B, Xing T-K, Wu G-S, Sun H-Y, et al Current Perspective in the Discovery of Anti-aging Agents from Natural Products. Natural products and bioprospecting. 2017;7(5):335–404. 10.1007/s13659-017-0135-9 . 28567542 PMC5655361

[6] Abdulla MA, Noor S, Wong K-H, Ali HM. Effect of Culinary-Medicinal Lion's Mane Mushroom, Hericium erinaceus (Bull.: Fr.) Pers. (Aphyllophoromycetideae), on Ethanol-Induced Gastric Ulcers in Rats. 2008;10(4):325–30. 10.1615/IntJMedMushr.v10.i4.40

[7] Cui F-J, Li Y-H, Zan X-Y, Yang Y, Sun W-J, Qian J-Y, et al Purification and partial characterization of a novel hemagglutinating glycoprotein from the cultured mycelia of Hericium erinaceus. Process Biochemistry. 2014;49(8):1362–9. 10.1016/j.procbio.2014.04.008.

[8] Ren Z, Qin T, Qiu F, Song Y, Lin D, Ma Y, et al Immunomodulatory effects of hydroxyethylated Hericium erinaceus polysaccharide on macrophages RAW264.7. Int J Biol Macromol. 2017;105(Pt 1):879–85. Epub 2017/07/22. 10.1016/j.ijbiomac.2017.07.104 . 28729219

[9] Yang BK, Park JB, Song CH. Hypolipidemic effect of an Exo-biopolymer produced from a submerged mycelial culture of Hericium erinaceus. Bioscience, biotechnology, and biochemistry. 2003;67(6):1292–8. Epub 2003/07/05. . 12843656 10.1271/bbb.67.1292

[10] Liang B, Guo Z, Xie F, Zhao A. Antihyperglycemic and antihyperlipidemic activities of aqueous extract of Hericium erinaceus in experimental diabetic rats. BMC complementary and alternative medicine. 2013;13:253–. 10.1186/1472-6882-13-253 . 24090482 PMC3852124

[11] Kim SP, Moon E, Nam SH, Friedman M. Hericium erinaceus Mushroom Extracts Protect Infected Mice against Salmonella Typhimurium-Induced Liver Damage and Mortality by Stimulation of Innate Immune Cells. Journal of agricultural and food chemistry. 2012;60(22):5590–6. 10.1021/jf300897w 22624604

[12] Kim SP, Nam SH, Friedman M. Hericium erinaceus (Lion's Mane) mushroom extracts inhibit metastasis of cancer cells to the lung in CT-26 colon cancer-tansplanted mice. Journal of agricultural and food chemistry. 2013;61(20):4898–904. Epub 2013/05/15. 10.1021/jf400916c . 23668749

[13] Han ZH, Ye JM, Wang GF. Evaluation of in vivo antioxidant activity of Hericium erinaceus polysaccharides. Int J Biol Macromol. 2013;52:66–71. Epub 2012/09/25. 10.1016/j.ijbiomac.2012.09.009 . 23000690

[14] Chang ST, Wasser SP. The role of culinary-medicinal mushrooms on human welfare with a pyramid model for human health. International journal of medicinal mushrooms. 2012;14(2):95–134. Epub 2012/04/18. . 22506573 10.1615/intjmedmushr.v14.i2.10

[15] Li I-C, Lee L-Y, Tzeng T-T, Chen W-P, Chen Y-P, Shiao Y-J, et al Neurohealth Properties of Hericium erinaceus Mycelia Enriched with Erinacines. Behavioural Neurology. 2018;2018:10 10.1155/2018/5802634 29951133 PMC5987239

[16] Tissenbaum HA, Guarente L. Model organisms as a guide to mammalian aging. Developmental cell. 2002;2(1):9–19. Epub 2002/01/10. . 11782310 10.1016/s1534-5807(01)00098-3

[17] Bitto A, Wang AM, Bennett CF, Kaeberlein M. Biochemical Genetic Pathways that Modulate Aging in Multiple Species. Cold Spring Harbor perspectives in medicine. 2015;5(11):10.1101/cshperspect.a025114 a. 10.1101/cshperspect.a025114 . 26525455 PMC4632857

[18] Pandey UB, Nichols CD. Human disease models in Drosophila melanogaster and the role of the fly in therapeutic drug discovery. Pharmacological reviews. 2011;63(2):411–36. 10.1124/pr.110.003293 . 21415126 PMC3082451

[19] Mouse Genome Sequencing C, Chinwalla AT, Cook LL, Delehaunty KD, Fewell GA, Fulton LA, et al Initial sequencing and comparative analysis of the mouse genome. Nature. 2002;420:520 https://www.nature.com/articles/nature01262#supplementary-information. 10.1038/nature01262 12466850

[20] Butterfield DA, Poon HF. The senescence-accelerated prone mouse (SAMP8): A model of age-related cognitive decline with relevance to alterations of the gene expression and protein abnormalities in Alzheimer's disease. Experimental gerontology. 2005;40(10):774–83. 10.1016/j.exger.2005.05.007 16026957

[21] Li IC, Chen YL, Lee LY, Chen WP, Tsai YT, Chen CC, et al Evaluation of the toxicological safety of erinacine A-enriched Hericium erinaceus in a 28-day oral feeding study in Sprague-Dawley rats. Food and chemical toxicology: an international journal published for the British Industrial Biological Research Association. 2014;70:61–7. Epub 2014/05/09. 10.1016/j.fct.2014.04.040 . 24810469

[22] Li IC, Chen YL, Chen WP, Lee LY, Tsai YT, Chen CC, et al Genotoxicity profile of erinacine A-enriched Hericium erinaceus mycelium. Toxicol Rep. 2014;1:1195–201. 10.1016/j.toxrep.2014.11.009 28962329 PMC5598247

[23] He Y, Jasper H. Studying aging in Drosophila. Methods (San Diego, Calif). 2014;68(1):129–33. Epub 04/18. 10.1016/j.ymeth.2014.04.008 . 24751824 PMC4066732

[24] Wu JJ, Liu J, Chen EB, Wang JJ, Cao L, Narayan N, et al Increased mammalian lifespan and a segmental and tissue-specific slowing of aging after genetic reduction of mTOR expression. Cell reports. 2013;4(5):913–20. Epub 2013/09/03. 10.1016/j.celrep.2013.07.030 ; PubMed Central PMCID: PMCPmc3784301. 23994476 PMC3784301

[25] Kim EK, Choi EJ. Compromised MAPK signaling in human diseases: an update. Archives of toxicology. 2015;89(6):867–82. Epub 2015/02/19. 10.1007/s00204-015-1472-2 . 25690731

[26] Lobo V, Patil A, Phatak A, Chandra N. Free radicals, antioxidants and functional foods: Impact on human health. Pharmacognosy reviews. 2010;4(8):118–26. 10.4103/0973-7847.70902 . 22228951 PMC3249911

[27] Barja G. Mitochondrial oxygen radical generation and leak: sites of production in states 4 and 3, organ specificity, and relation to aging and longevity. Journal of bioenergetics and biomembranes. 1999;31(4):347–66. Epub 2000/02/09. . 10665525 10.1023/a:1005427919188

[28] Lee KF, Chen JH, Teng CC, Shen CH, Hsieh MC, Lu CC, et al Protective effects of Hericium erinaceus mycelium and its isolated erinacine A against ischemia-injury-induced neuronal cell death via the inhibition of iNOS/p38 MAPK and nitrotyrosine. International journal of molecular sciences. 2014;15(9):15073–89. 10.3390/ijms150915073 25167134 PMC4200813

[29] Kuo HC, Lu CC, Shen CH, Tung SY, Hsieh MC, Lee KC, et al Hericium erinaceus mycelium and its isolated erinacine A protection from MPTP-induced neurotoxicity through the ER stress, triggering an apoptosis cascade. J Transl Med. 2016;14:78 10.1186/s12967-016-0831-y 26988860 PMC4797317

[30] Chiu C-H, Chyau C-C, Chen C-C, Lee L-Y, Chen W-P, Liu J-L, et al Erinacine A-Enriched Hericium erinaceus Mycelium Produces Antidepressant-Like Effects through Modulating BDNF/PI3K/Akt/GSK-3β Signaling in Mice. International journal of molecular sciences. 2018;19(2):341 10.3390/ijms19020341 29364170 PMC5855563

[31] Zelko IN, Mariani TJ, Folz RJ. Superoxide dismutase multigene family: a comparison of the CuZn-SOD (SOD1), Mn-SOD (SOD2), and EC-SOD (SOD3) gene structures, evolution, and expression. Free Radic Biol Med. 2002;33(3):337–49. Epub 2002/07/20. . 12126755 10.1016/s0891-5849(02)00905-x

[32] Yiin SJ, Lin TH, Shih TS. Lipid peroxidation in workers exposed to manganese. Scandinavian journal of work, environment & health. 1996;22(5):381–6. Epub 1996/10/01. . 8923613 10.5271/sjweh.158

[33] Shih PH, Yen GC. Differential expressions of antioxidant status in aging rats: the role of transcriptional factor Nrf2 and MAPK signaling pathway. Biogerontology. 2007;8(2):71–80. Epub 2006/07/20. 10.1007/s10522-006-9033-y . 16850181

[34] Gredilla R, Sanz A, Lopez-Torres M, Barja G. Caloric restriction decreases mitochondrial free radical generation at complex I and lowers oxidative damage to mitochondrial DNA in the rat heart. Faseb j. 2001;15(9):1589–91. Epub 2001/06/28. . 11427495 10.1096/fj.00-0764fje

[35] Sanz A, Fernandez-Ayala DJ, Stefanatos RK, Jacobs HT. Mitochondrial ROS production correlates with, but does not directly regulate lifespan in Drosophila. Aging. 2010;2(4):200–23. Epub 2010/05/11. 10.18632/aging.100137 ; PubMed Central PMCID: PMCPmc2880708. 20453260 PMC2880708

[36] Gruber J, Schaffer S, Halliwell B. The mitochondrial free radical theory of ageing—where do we stand? Frontiers in bioscience: a journal and virtual library. 2008;13:6554–79. Epub 2008/05/30. . 18508680 10.2741/3174

[37] Harman D. Aging: A Theory Based on Free Radical and Radiation Chemistry. Journal of Gerontology. 1956;11(3):298–300. 10.1093/geronj/11.3.298 13332224

[38] Schriner SE, Linford NJ, Martin GM, Treuting P, Ogburn CE, Emond M, et al Extension of murine life span by overexpression of catalase targeted to mitochondria. Science. 2005;308(5730):1909–11. Epub 2005/05/10. 10.1126/science.1106653 . 15879174

[39] Wanagat J, Dai D-F, Rabinovitch P. Mitochondrial oxidative stress and mammalian healthspan. Mechanisms of Ageing and Development. 2010;131(7):527–35. 10.1016/j.mad.2010.06.002. 20566356 PMC2933331

[40] Lee K-C, Kuo H-C, Shen C-H, Lu C-C, Huang W-S, Hsieh M-C, et al A proteomics approach to identifying novel protein targets involved in erinacine A-mediated inhibition of colorectal cancer cells' aggressiveness. Journal of cellular and molecular medicine. 2017;21(3):588–99. Epub 10/06. 10.1111/jcmm.13004 . 27709782 PMC5323879

[41] Redman LM, Ravussin E. Caloric restriction in humans: impact on physiological, psychological, and behavioral outcomes. Antioxidants & redox signaling. 2011;14(2):275–87. 10.1089/ars.2010.3253 . 20518700 PMC3014770

[42] Lee GD, Wilson MA, Zhu M, Wolkow CA, de Cabo R, Ingram DK, et al Dietary deprivation extends lifespan in Caenorhabditis elegans. Aging cell. 2006;5(6):515–24. Epub 2006/11/14. 10.1111/j.1474-9726.2006.00241.x ; PubMed Central PMCID: PMCPmc2546582. 17096674 PMC2546582

[43] Jafari M, Rose MR. Rules for the use of model organisms in antiaging pharmacology. Aging cell. 2006;5(1):17–22. 10.1111/j.1474-9726.2006.00195.x 16441839

[44] Matyi S, Jackson J, Garrett K, Deepa SS, Unnikrishnan A. The effect of different levels of dietary restriction on glucose homeostasis and metabolic memory. GeroScience. 2018;40(2):139–49. 10.1007/s11357-018-0011-5 29455275 PMC5964050

[45] Li IC, Chen WP, Chen YP, Lee LY, Tsai YT, Chen CC. Acute and developmental toxicity assessment of erincine A-enriched Hericium erinaceus mycelia in Sprague-Dawley rats. Drug and chemical toxicology. 2018:1–6. Epub 2018/01/24. 10.1080/01480545.2017.1381110 . 29359595

